# The impact of public health insurance on healthcare utilisation in Indonesia: evidence from panel data

**DOI:** 10.1007/s00038-019-01215-2

**Published:** 2019-02-08

**Authors:** Darius Erlangga, Shehzad Ali, Karen Bloor

**Affiliations:** Department of Health Sciences, University of York, York, UK

**Keywords:** Health insurance, Developing countries, Utilisation, Policy evaluation

## Abstract

**Objectives:**

This study is the first rigorous evaluation of the impact of Jaminan Kesehatan Nasional (JKN) on improving access to outpatient and inpatient care, utilising longitudinal data from the Indonesian Family Life Survey.

**Methods:**

Two treatment groups were identified: a contributory group (*N* = 982), who paid the premium voluntarily, and a subsidised group (*N* = 2503), paid by government. Each group was compared with the uninsured group (*N* = 8576). Propensity score matching combined with difference-in-difference approaches was used to estimate the causal effect of the JKN programme.

**Results:**

The results found that JKN increased the probability of inpatient admission for the contributory and subsidised groups by 8.2% (95% CI 5.9–10.5%) and 1.8% (95% CI 0.7–2.82%), respectively. The contributory group had an increase in probability of an outpatient visit of 7.9% (95% CI 4.3–11.4%).

**Conclusions:**

The JKN programme has increased the utilisation of outpatient and inpatient care in the contributory group. Those with subsidised insurance have an increase in access to inpatient facilities only, and this is of a smaller magnitude. Hence, while JKN has improved average utilisation, inequity in access to both outpatient and inpatient care may remain.

**Electronic supplementary material:**

The online version of this article (10.1007/s00038-019-01215-2) contains supplementary material, which is available to authorized users.

## Introduction

Universal health coverage (UHC) is a key health policy concern in most low- and middle-income countries (LMICs) (Lagomarsino et al. 2012; World Health Organization 2014; Maeda et al. 2014). The inclusion of UHC in the health section of the United Nations Sustainable Development Goals (SDGs) has created renewed momentum for national health insurance schemes (United Nations 2018). Therefore, it is important for countries introducing or expanding health insurance to learn from experience of other countries, and this study contributes to this important evidence base.

By mid-2018, nearly 186 million individuals in Indonesia (76% of the total population) were covered by *Jaminan Kesehatan Nasional* (JKN), one of the largest single payer social health insurance programmes in the world (Pinto et al. 2016; BPJS Kesehatan 2017). Considering the low coverage of private health insurance (1.5%), it is estimated that 22.5% of Indonesian population is still uninsured (Mahendradhata et al. 2017). Introduced in January 2014, the JKN programme unified several previously fragmented public health insurance, including *Askes* (which covered public formal sector employees), *Jamsostek* (private formal sector employees), and *Jamkesmas* (the poorest population). In general, there are two big groups of JKN enrollees: (1) *the subsidised group* or *Penerima Bantuan Iuran (PBI)/Contribution Assistance Recipients* including the poor population and disabled individuals, and (2) *the contributory group* consisting of *Peserta Pekerja Penerima Upah (PPU)/*salaried employees (government and private), *Peserta Pekerja Bukan Penerima Upah (PBPU)/*non-salaried workers, and *Peserta Bukan Pekerja/*non-workers. While the salaried employees are required to contribute a certain percentage of their salaries, the other two groups are required to contribute by paying a fixed amount of premium based on their chosen inpatient ward class (Kesehatan 2017). The difference in inpatient ward classes mostly determines the amount of non-medical facilities, but all patients should receive a similar quality of medical services regardless of the class.

A descriptive analysis from the Indonesian socioeconomic survey (i.e. SUSENAS) showed an increasing trend of utilisation for both outpatient and inpatient care amongst the JKN enrollees compared to the uninsured in 2016. However, this finding is likely to be sensitive to insurance selection bias as the survey is not randomised, and there is no control for previous insurance status prior to the introduction of JKN in 2014 (Statistics Indonesia 2018). Previous studies have evaluated earlier forms of health insurance using different datasets and approaches, with mixed findings. Johar evaluated the health cards programme introduced in 2000 and found that it did not increase outpatient utilisation due to the inelastic demand amongst the recipients (Johar 2009). Hidayat and Pokhrel analysed the impact of Askes and Jamsostek and found a positive outpatient utilisation effect especially on private facilities (Hidayat and Pokhrel 2010). Sparrow et al. evaluated the health insurance programme for poor people (Askeskin) and found positive utilisation effects on outpatient care (Sparrow et al. 2013). Lastly, Vidyattama et al. evaluated Askeskin using different datasets and found a positive effect on the probability of utilising outpatient care (Vidyattama et al. 2014).

Recent longitudinal data from the Indonesia Family Life Survey (IFLS) in 2014 provide an opportunity to conduct the impact evaluation of the JKN programme. In this study, we examine whether the implementation of Indonesia’s JKN programme improved access to health care, measured in terms of utilisation, for its enrollees. Subgroup analysis explores differences in impact between socio-economic groups, urban/rural areas, and the availability of healthcare facilities. Importantly, we also distinguish two types of JKN enrollees, subsidised and contributory group, as we believe both groups have different characteristics which may influence both decision to get insured and seek care. Our study is also the first empirical study exploring utilisation of inpatient care in Indonesia, which is underreported in most Indonesian health insurance studies.

## Methods

### Study population and data source

The main data were obtained from the Indonesia Family Life Survey (IFLS) 2007 and 2014. IFLS is a longitudinal survey of socio-economic characteristics and population health; the survey is based on a sample of households living in 13 of the country’s 27 provinces in 1993. The selected provinces were chosen to maximise representation of the population (83% of the Indonesian population) and be cost-effective to survey given the vast area and difficult terrain of the country (Strauss et al. 2016). All IFLS data are publicly available. The JKN programme began in January 2014 and implemented nationally. IFLS 2014 was conducted between September 2014–March 2015, which means that IFLS 2007 data can be treated as the baseline and 2014 data as the follow-up, thereby allowing panel data analysis. Response rate in IFLS 2007 was 93.6% (Strauss et al. 2016). There were 29,014 adults who completed individual questionnaires in 2007, but only 22,711 individuals completed the same questionnaires in 2014, yielding an attrition rate of 21.73%. The reasons for non-completion amongst the 6303 individuals are depicted in Online Resource 1.

### Treatment and control groups

In order to estimate a causal effect from a before and after study, both the treated and control groups must be uninsured in 2007. Furthermore, the treated group must have no other insurance than the JKN programme, including previous insurance with Askes, Jamsostek, or private insurance, to prevent the spill-over effect. Out of 22,711 individuals, we excluded 10,650 individuals following those two criteria. Thus, the following treatment and control groups were defined for this analysis:

1. JKN contributory group (*N* = 982): individuals who were uninsured in 2007 but then enrolled voluntarily in 2014. This group may represent self-employed individuals or people who worked in the informal sector, but they were not categorised as poor.
2. JKN subsidised group (*N* = 2503): individuals who were uninsured in 2007 but qualified for subsidised JKN premiums in 2014. This group is qualified for subsidised premiums based on a proxy means test defined by the government.
3. Uninsured group (*N* = 8576): individuals who were uninsured in 2007 and remained uninsured in 2014.

In this analysis, each of the contributory and subsidised group was compared to the uninsured, as a control group, separately.

### Outcome and control variables

The outcome variables were use of outpatient care in the last 4 weeks and use of inpatient care in the last 12 months. Longer period for inpatient care was chosen as this type of care is rarely used compared to outpatient care (Bhandari and Wagner 2006). For both types of care, two related outcomes were defined: a binary variable taking the value of one if the respondent reported seeking care, and a continuous variable which records the frequency of visits. The number of visits was also differentiated based on type of facilities: public or private.

We followed Andersen’s behavioural model in choosing control variables for our model. We controlled for age, gender, marital status, urban/rural residence, education level, and socio-economic status as those are predisposing characteristics and enabling factors that influence people’s decision to seek care (Aday and Andersen 1974). Assets index was chosen as a proxy measure of socio-economic status when neither income nor expenditure data are available (Filmer and Pritchett 2001; Sahn and Stifel 2003). Information on the asset index was based on a number of input variables, including durable assets and dwelling characteristics. Principal components analysis (PCA) was employed in creating the asset index (Vyas and Kumaranayake 2006). The Cronbach’s alpha for the assets index is 0.78 which lies within the acceptable range of 0.7–0.8 indicating a good internal consistency (Bland and Altman 1997). In this analysis, we also included three health status variables to capture the evaluated need that may prompt individuals to seek care (Andersen 1995): the number of acute conditions, the number of chronic conditions, and the presence of disability. Medical conditions included in the construction of health status variable are explained in Online Resource 2. Multiple health conditions are associated with increased utilisation hence the inclusion of a number of conditions (Palladino et al. 2016). We also included the availability of healthcare facilities in the community area as a density variable, separated into primary care facilities for outpatient care and hospitals for inpatient care, to control for supply of health care. Binary variables for each IFLS province are also included to capture unobserved time-fixed effect that may correlate with the demand and supply of care in the area (Gravelle et al. 2003). We also included a binary variable indicating the recipients of unconditional cash transfer as it may influence individual’s decision to seek care by increasing household income temporarily (Sparrow et al. 2013).

An important consideration when estimating the impact of JKN is the insurance selection bias. A decision to enrol in health insurance may not be random, i.e. it may be correlated with the outcome of health insurance (Cutler and Zeckhauser 1998). Hence, any observed and unobserved factors influencing the participation decision can potentially introduce bias in our estimation model. To overcome this, we utilised the panel structure of IFLS data by combining a difference-in-differences (DID) approach with propensity score matching (PSM). We accounted for potential bias due to observable factors using PSM which balances the observed characteristics of the insured and uninsured groups. An attractive feature of PSM compared to regression type estimators is its nonparametric nature because PSM assumes a flexible functional form to estimate the outcome model (Rosenbaum and Rubin 1983). A better statistical balance between treatment and control group after matching based on the estimated propensity score is more important than finding the appropriate model for the outcome variables (Wagstaff et al. 2009).

To implement PSM, a logit model was estimated for log odds of enrolment in JKN programme in 2014 using control variables in 2007, ensuring the exogeneity of the observables (Caliendo and Kopeinig 2008). Based on this model, the propensity score was predicted for each individual for both contributory and subsidised groups separately. In addition, we included the sample weight to achieve unbiased treatment effect estimates generalisable to the original survey target population (Dugoff et al. 2014).

Kernel matching was chosen as the matching algorithm with a choice of calliper of bandwidth equal to 0.2 of the standard deviation of the logit of the propensity score (Austin 2011). Standard errors were calculated by bootstrapping to allow for an estimation of the sampling variance of estimated propensity score parameters (Caliendo and Kopeinig 2008). We also generated the histograms of the propensity scores after matching to check overlap and region of common support, and the scatterplot of the standardised differences vs residual variance ratios to check covariate imbalance before and after matching (Leuven and Sianesi 2003).

Next, we used DID to account for any time-fixed unobservable factors that may bias our estimates (Heckman et al. 1998; Wagstaff et al. 2009). An important assumption in DID analysis is parallel trend assumption which assumes that the outcome for both insured and uninsured groups follow a similar trend before the introduction of health insurance. To test this assumption, we performed a placebo test by estimating the impact of JKN on the DID estimates from IFLS 2000 and 2007 (Angrist and Pischke 2008). If this assumption is valid, then the treatment variable should not have any statistically significant effect on past outcomes at 5% level.

While DID is able to eliminate time-fixed unobservable factors, the unobservable bias due to time-varying unobservable factors persists. To assess this bias, we calculated the Rosenbaum bounds for the treatment effects. This test gives an indication of the extent of this bias required to undermine interpretation of the propensity score estimates (Rosenbaum 2002). The objective is to determine the smallest value of bias that will change the *p* value of the relationship between treatment and outcomes to a non-significant level (Liu et al. 2013). All analyses were performed using Stata v14.

## Results

### Descriptive statistics

Table 1 presents a descriptive table of the outcomes and control variables for each group. Compared to the uninsured, both insured groups had higher proportion and frequency of utilisation of outpatient and inpatient care in both years, except for higher proportion and total inpatient visits for the uninsured compared to the subsidised group in 2007. These results indicated the possibility of an insurance selection effect, as the insured groups were observed to have a higher probability or level before the reform was introduced.

**Table 1:** Summary statistics for outcome and control variables by insurance status, Indonesia, 2007 and 2014

Variables	2007			2014		
	Uninsured (*N* = 8564)	Contributory (*N* = 975)	Subsidised (*N* = 2495)	Uninsured (*N* = 8564)	Contributory (*N* = 975)	Subsidised (*N* = 2495)
Outcome variables						
Proportion of having outpatient visits (%)	12	14.4	13.2	14.5	23.4	17.4
Number of outpatient visits (all)	0.16	0.25	0.19	0.23	0.44	0.32
Number of outpatient visits (public)	0.04	0.05	0.07	0.07	0.18	0.16
Number of outpatient visits (private)	0.12	0.20	0.12	0.16	0.26	0.16
Proportion of having inpatient visits (%)	2	3.3	1.7	2.6	11.2	4.2
Number of inpatient visits (all)	0.022	0.036	0.018	0.037	0.149	0.058
Number of inpatient visits (public)	0.012	0.015	0.012	0.021	0.088	0.042
Number of inpatient visits (private)	0.010	0.021	0.006	0.016	0.061	0.016
Control variables						
Age (year)	37	33	38	43	40	44
Male (%)	46	42	45	46	42	45
Single (%)	19	24	15	9	10	6
Married (%)	73	72	77	79	82	81
Divorced/widowed (%)	9	4	8	13	9	12
Urban (%)	41	71	44	41	71	44
Primary education (%)	41	22	50	41	21	49
Secondary education (%)	44	61	38	43	60	38
College (%)	2	6	1	2	5	1
Higher education (%)	3	8	1	6	13	2
No education (%)	9	3	9	8	2	9
Poorest—lowest quintile* (%)	20	9	33	20	7	32
Richest—highest quintile* (%)	16	35	5	17	40	7
No. of acute conditions	2	2.4	2	3	4	4
No. of chronic conditions	0.16	0.15	0.14	0.32	0.38	0.31
Any disability (%)	0.9	1.4	0.3	8	12	7
Density of outpatient health facilities**	0.2	0.6	0.1	0.2	0.6	0.1
Density of inpatient health facilities**	0.04	0.1	0.02	0.05	0.2	0.03
Recipient of unconditional cash transfer (%)	16	13	35	1	11.2	53

*Quintiles were determined based on assets index

**Density variables were derived from number of facilities divided by the village/township size in hectare (1 hectare = 10,000 m^2^)

Looking at the control variables, individuals covered by the JKN contributory scheme were younger, more likely to live in an urban area, wealthier, more likely to have completed higher education, and more likely to live in an area with more health facilities compared to the uninsured. Meanwhile, individuals with JKN subsidised were poorer, less likely to finish higher education, more likely to receive cash transfers, and living in an area with fewer health facilities compared to the uninsured. Overall, this comparison confirms our suspicion that the JKN contributory and subsidised groups have different characteristics that may influence the decision to get insured and seek care.

### Impact estimates

**Fig. 1:**
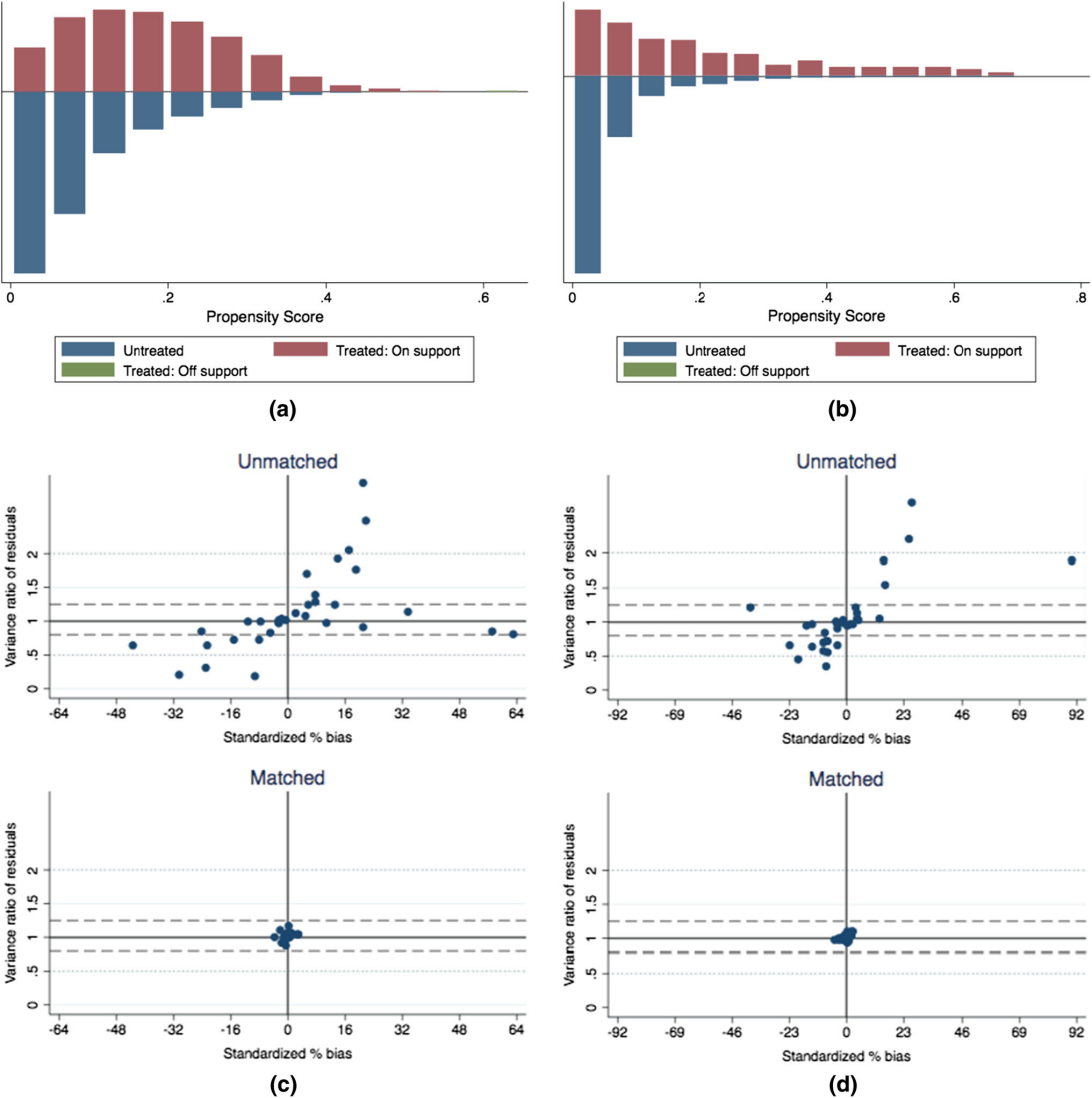
Common support and bias balance after kernel matching for both insured and uninsured population, Indonesia, 2007–2014. All figures were produced by Stata v14. **a** Shows support between treated and untreated for the contributory group, whereas **b** is for the subsidised group. Each bar represents the density of observations from the insured and uninsured. Common support assumption is satisfied when there are enough untreated observations paired with the treated within the same propensity score range. **c** and **d** Show the reduced bias before and after matching for the contributory and subsidised group, respectively. It is desirable to have both standardised percent bias and variance ratio of residuals as low as possible (near zero)

Figure 1a and b shows the histograms for the propensity scores after matching. Despite its skewed distribution, there are ample overlaps between the treated and the control group implying that the matching has successfully retained adequate samples to avoid attrition bias from the cases of off-support. Figure 1c and d shows that after matching, the standardised percentage of bias across covariates has been reduced to near zero.

Table 2 reports results of the PSM-DID analysis of outpatient care while Table 3 shows findings for inpatient care. Based on Tables 2 and 3, the contributory group had 7.9 per cent (95% CI 4.3–11.4%) and 8.2 per cent (95% CI 5.9–10.5%) higher probabilities of using outpatient and inpatient care, respectively, compared to the uninsured. In addition, the contributory group had 0.16 (95% CI 0.05–0.27) more outpatient visits per person per month and 0.1 (95% CI 0.08–0.14) more inpatient visits per person per year compared to the uninsured. This higher number of total visits was likely to occur in public facilities.

**Table 2:** Impact of the Jaminan Kesehatan Nasional (JKN) programme on outpatient utilisation for both contributory and subsidised groups, stratified by asset index quintiles, urban/rural area, and density of healthcare facilities, Indonesia, 2007 and 2014

	Probability of having outpatient visits	Number of outpatient visits (all)	Number of outpatient visits (public)	Number of outpatient visits (private)
*Panel A: contributory group*				
Overall	0.079***	0.158***	0.115***	0.043
	(0.018)	(0.057)	(0.022)	(0.047)
Quintile 1 (poorest)	− 0.024	− 0.018	0.052	− 0.070
	(0.063)	(0.112)	(0.056)	(0.091)
Quintile 2	0.113**	0.312	0.173*	0.139
	(0.056)	(0.194)	(0.099)	(0.142)
Quintile 3	0.106***	0.172	0.176***	− 0.005
	(0.031)	(0.279)	(0.065)	(0.279)
Quintile 4	0.088**	0.081	0.060*	0.021
	(0.039)	(0.073)	(0.033)	(0.066)
Quintile 5 (richest)	0.083**	0.207**	0.126***	0.081
	(0.038)	(0.081)	(0.037)	(0.066)
Urban	0.085***	0.146**	0.119***	0.026
	(0.021)	(0.066)	(0.025)	(0.060)
Rural	0.068**	0.199*	0.109***	0.090
	(0.032)	(0.111)	(0.031)	(0.093)
Low density^a^	0.016	0.097	0.046	0.051
	(0.038)	(0.153)	(0.040)	(0.165)
High density^a^	0.035	0.067	0.148**	− 0.081)
	(0.031)	(0.103)	(0.060)	(0.099)
*Panel B: subsidised group*				
Overall	0.019	0.063***	0.059***	0.004
	(0.018)	(0.024)	(0.018)	(0.016)
Quintile 1 (poorest)	− 0.011	0.013	0.009	0.004
	(0.021)	(0.046)	(0.039)	(0.023)
Quintile 2	0.069***	0.126**	0.112***	0.015
	(0.027)	(0.060)	(0.037)	(0.034)
Quintile 3	0.006	0.056	0.087*	− 0.031
	(0.020)	(0.047)	(0.045)	(0.033)
Quintile 4	0.013	0.047	0.066*	− 0.020
	(0.024)	(0.044)	(0.034)	(0.033)
Quintile 5 (richest)	0.093*	0.180*	0.044	0.136*
	(0.054)	(0.101)	(0.060)	(0.081)
Urban	0.032	0.112***	0.114***	− 0.002
	(0.021)	(0.038)	(0.028)	(0.027)
Rural	0.011	0.025	0.019	0.006
	(0.013)	(0.037)	(0.024)	(0.029)
Low density^a^	0.016	0.068	0.017	0.051
	(0.020)	(0.037)	(0.031)	(0.028)
High density^a^	0.048	0.155**	0.134***	0.021
	(0.032)	(0.060)	(0.032)	(0.049)

^a^The samples were first sorted from the lowest to the highest based on the density variables and then divided into four equal group (quartiles). The first and fourth quartiles become the low density and high density, respectively

The reported standard errors in parentheses were calculated by bootstrapping with 200 replications. Quintiles were determined based on assets index in 2007. Significance: *p < 0.1; **p < 0.05; ***p < 0.01

**Table 3:** Impact of the Jaminan Kesehatan Nasional (JKN) programme on inpatient utilisation for both the contributory and subsidised groups, by asset index quintiles, urban/rural area, and density of healthcare facilities, Indonesia, 2007 and 2014

	Probability of having inpatient visits	Number of inpatient visits (all)	Number of inpatient visits (public)	Number of inpatient visits (private)
*Panel A: contributory group*				
Overall	0.082***	0.109***	0.073***	0.036***
	(0.012)	(0.015)	(0.010)	(0.013)
Quintile 1 (poorest)	0.041	0.046	0.006	0.040
	(0.047)	(0.056)	(0.035)	(0.029)
Quintile 2	0.081**	0.184***	0.117**	0.067
	(0.029)	(0.061)	(0.048)	(0.048)
Quintile 3	0.099***	0.113**	0.083**	0.030
	(0.034)	(0.050)	(0.038)	(0.025)
Quintile 4	0.095***	0.126***	0.090**	0.036**
	(0.029)	(0.041)	(0.036)	(0.015)
Quintile 5 (richest)	0.071***	0.080***	0.054***	0.026
	(0.019)	(0.025)	(0.012)	(0.023)
Urban	0.097***	0.128***	0.082***	0.046***
	(0.015)	(0.021)	(0.016)	(0.015)
Rural	0.044**	0.063**	0.051***	0.013
	(0.018)	(0.031)	(0.015)	(0.020)
Low density^a^	0.025	0.050	0.038	0.013
	(0.022)	(0.050)	(0.038)	(0.013)
High density^a^	0.103***	0.176***	0.105***	0.076***
	(0.022)	(0.039)	(0.025)	(0.076)
*Panel B: subsidised group*				
Overall	0.017***	0.023***	0.018***	0.005
	(0.005)	(0.009)	(0.007)	(0.005)
Quintile 1 (poorest)	0.015	0.010	0.012	− 0.002
	(0.010)	(0.012)	(0.009)	(0.006)
Quintile 2	− 0.004	− 0.001	0.010	− 0.011
	(0.013)	(0.014)	(0.014)	(0.006)
Quintile 3	0.032***	0.042**	0.033*	0.009
	(0.011)	(0.021)	(0.019)	(0.008)
Quintile 4	0.030**	0.049*	0.039	0.010
	(0.014)	(0.028)	(0.027)	(0.007)
Quintile 5 (richest)	0.017	0.043	0.009	0.034
	(0.023)	(0.049)	(0.029)	(0.047)
Urban	0.016*	0.026*	0.019	0.007
	(0.009)	(0.016)	(0.015)	(0.008)
Rural	0.017**	0.019*	0.018**	0.001
	(0.007)	(0.011)	(0.009)	(0.005)
Low density^a^	0.016	0.012	− 0.001	0.008
	(0.011)	(0.017)	(0.012)	(0.010)
High density^a^	0.030***	0.047***	0.029***	0.021
	(0.013)	(0.018)	(0.010)	(0.017)

^a^The samples were first sorted from the lowest to the highest based on the density variables and then divided into four equal group (quartiles). The first and fourth quartiles become the low density and high density, respectively

The reported standard errors in parentheses were calculated by bootstrapping with 200 replications. Quintiles were determined based on assets index in 2007. Significance: *p < 0.1; **p < 0.05; ***p < 0.01

Looking at the second panel of both Tables 2 and 3, it appears that the JKN programme increased the probability of seeking care at outpatient facilities amongst the subsidised group by 2 per cent (95% CI − 0.4 to 4.3%). Meanwhile, the subsidised group also increased their probability of having any inpatient visit by 1.76 per cent (95% CI 0.7–2.8%) compared to the uninsured. In addition, the JKN subsidised group spent more number of visits to both outpatient and inpatient care compared to the uninsured.

Tables 2 and 3 also demonstrate the impact of JKN programme stratified by quintiles of the asset index. The impact on the contributory group was observed across all quintiles, except the poorest (first quintile). Meanwhile, the effects on the subsidised group showed a different pattern: increased outpatient utilisation was higher in the second quintile, but the effect on inpatient utilisation was stronger amongst the third and fourth quintiles. No effect was observed amongst the poorest quintile.

The impact estimates were also stratified by urban and rural area. Amongst the contributory group, enrollees from both rural and urban areas showed a similar pattern of positive and significant effect on both outpatient and inpatient utilisation. Subsidised individuals living in rural areas showed a positive impact on inpatient utilisation, whereas those living in urban areas showed a positive impact only on the frequency of outpatient utilisation in public facilities.

Table 4 also demonstrates the heterogeneity of the JKN effect by supply-side factors, measured by the density of healthcare facilities. The calculation of density variables was done separately for outpatient and inpatient care. Then, we sorted the samples from the lowest to the highest based on the density variables and divided the samples into four equal group (quartiles). We compared the effect on the lowest density (first quartile) and the highest quartile (fourth quartile). Almost no significant effect was observed in the area with a low density of healthcare facilities. In the high-density area, however, the effect on inpatient visits was large and significant for both the contributory and subsidised groups. This further confirms the suggestion that the effect of health insurance can only be realised given the availability of nearby healthcare facilities.

**Table 4:** Rosenbaum bounds analyses for the effect of the Jaminan Kesehatan Nasional (JKN) programme on both contributory and subsidised groups (the comparator for each group is the uninsured), Indonesia, 2000–2014

	Rosenbaum bounds*		Placebo test**			
	Contributory	Subsidised	Contributory		Subsidised	
			Treatment effect	*p* value	Treatment effect	*p* value
Outpatient						
Probability of any outpatient care	1.5	1.1	− 0.62%	0.62	0.76%	0.59
Number of outpatient visits (total)	1.1	1.1	− 0.007	0.73	0.018	0.45
Number of outpatient visits (public)	1.1	1.1	0.010	0.50	0.021	0.17
Number of outpatient visits (private)	1.1	1.2	− 0.017	0.28	− 0.003	0.87
Inpatient						
Probability of any inpatient care	3	1.5	− 0.64%	0.17	− 0.95%	0.08
Number of inpatient visits (total)	1.5	1.1	− 0.007	0.20	− 0.010	0.11
Number of inpatient visits (public)	1.7	1.1	− 0.002	0.44	− 0.003	0.40
Number of inpatient visits (private)	1.7	1.1	− 0.003	0.41	− 0.007	0.06

*Rosenbaum bounds column shows the coefficient representing the minimum effect of the unobserved time-varying factors would need to have to bias our treatment effect

**Parallel trend assumption can be upheld if the treatment effect of the placebo test shows no significant effect with assumed type-1 error taken at 5% level

To ensure the validity of our results, we conducted several robustness checks. Firstly, we checked the potential influence of the unobserved time-varying confounders by calculating Rosenbaum bounds (Table 4). The effect on the probability of utilising inpatient care looks more stable than the effect on outpatient care. The effect on the JKN contributory group is only sensitive to a bias that would triple the effect of insurance on probability of seeking inpatient care, whereas the subsidised group has a lower threshold. All frequency variables, however, are quite sensitive to unobserved time-varying confounders.

Secondly, we ran a placebo regression to test the parallel trend assumption by using data from IFLS 2000 and 2007 (Table 4). Parallel trend assumption is valid if none of the outcomes in this placebo test are significant. From Table 4, it appears that none of the outcome variables shows any significant effect, taken as a *p* value equal to or less than 0.05. The PSM-DID model therefore passed the parallel trend assumption.

Thirdly, we also checked the robustness of our impact estimates by different calliper of kernel matching (see online resources 3). It is shown in Online Resources 3 that overall our impact estimates for both groups are not sensitive to the size of the bandwidth for calculating the distance in kernel matching. The magnitude and the significance of the estimates seemed stable even at bandwidth 0.001.

## Discussion

This study has analysed the impact of JKN programme on access to care measured by individual’s healthcare utilisation. This study’s findings suggest that the JKN programme has increased the probability of individuals seeking outpatient and inpatient care. This impact is stronger amongst the contributory group, which likely comes from the wealthier and more educated population. This finding is consistent with evidence from other countries (Nguyen 2012; Robyn et al. 2012; Miller et al. 2013; Bernal et al. 2017). The impact on frequency of visits, however, is more sensitive to unobserved time-varying variables indicating that our estimated treatment effects may overestimate the true treatment effect on frequency of visits. We also found a marginal increase in utilisation amongst the subsidised group but this impact is more sensitive to unobserved time-varying factors. It is also likely that any effect amongst the subsidised group was picking up lagged effects from the introduction of Jamkesmas in 2008 that was targeted to poor population. Overall, we found limited evidence to support the benefit of the JKN programme for the subsidised group.

Our study also showed that the majority of uninsured individuals in 2007 remained uninsured in 2014 implying slow JKN enrolment process. The contributory group represents self-selected participation in the JKN programme, while the subsidised group has limited power to determine their eligibility. Therefore, the success of the JKN programme hinges on factors that influence people’s decision to join the JKN contributory scheme. People are more likely to enrol in health insurance if they are more likely to use them, referred to as adverse selection in economic literature (Cutler and Zeckhauser 1998). Individuals themselves have the best knowledge of whether the benefit of insurance exceeds the cost, which determines whether or not people decide to get insured (Kahneman et al. 1991; Schneider 2004). The contributory group may also be more proactive in seeking information and treatment and be more aware of the benefits from the JKN programme (considered a very comprehensive system) due to having a higher level of education. Recent evidence from Indonesia revealed that insurance premiums are not the major deterrent factor in JKN enrolment, but that patients are more likely to be influenced by the availability of health services and a lack of insurance literacy (Dartanto et al. 2016).

It appears that most health insurance studies in Indonesia seem to avoid analysing the impact on inpatient care due to the fear of low statistical power associated with inpatient care. In this study, this low power concern does not deter finding a significant effect as 1064 out of 22,708 individuals reported any inpatient visit in any formal healthcare facilities. Rather, we showed that the impact of the JKN programme was relatively larger on inpatient care compared to outpatient care. Since inpatient care is generally more expensive, and the JKN programme offers comprehensive benefits including hospitalisation in both public and contracted private hospitals, individuals are more likely to enrol, particularly if they consider themselves as a high-risk individual.

Despite our effort to control for the selection bias by combining PSM and DID, this study still has several limitations. First, some supply factors have not been controlled adequately, such as the distance to the nearest facilities or the qualities of health workers. Nevertheless, this study has attempted to control for supply factors by including the density of health facilities available in the village/township in which the respondents were currently living. Second, IFLS is not representative of all Indonesian provinces, and thus, it cannot produce a national estimate. IFLS excluded most eastern Indonesian provinces, which are considered underdeveloped compared to their western counterparts. Another data set that encompasses all Indonesian provinces is available [e.g. Indonesian Socioeconomic Survey (SUSENAS)], but it does not provide adequate health insurance status information or on health utilisation prior to 2014. Also, IFLS is the only Indonesian panel data set available to evaluate the JKN, hence its inclusion in this study. Considering IFLS provinces are more developed than the non-IFLS provinces, our findings may show the upper limit of the true impact. It is likely that JKN has much rather limited impact in the non-IFLS provinces due to lack of health facilities in underdeveloped provinces, but the extent of it is another empirical question.

While it is encouraging to observe the positive impact on both subsidised and contributory groups, the greater effect on the contributory group indicates a potential adverse selection effect amongst the more affluent population. Given the fact that the subsidised group accounts for the largest proportion of the insured population, their subsidies paid by the government also takes up more of the JKN budget. This implies a potential inequity in how government subsidies are being targeted in the sense that the poor did not receive the benefit from the subsidy.

This inequity issue is exacerbated by the fact that the JKN effect is much stronger in the area with higher density of healthcare facilities. Since the subsidised group is more likely to live in rural area with limited healthcare facilities, we can expect to observe limited effect of insurance in removing barrier to access of care. Insurance may ease the financial barriers associated with the fees for medical treatment (i.e. affordability) but may not be adequate to remove other barriers to access, such as the cost of transportation (accessibility) or the availability of primary clinics and hospitals (Penchansky and Thomas 1981). Improving access to care amongst individuals in the rural and remote area is still a big homework for the Indonesian government; the problem that cannot be solved only by the introduction of public health insurance for all.

Following this potential inequity, it might be appealing to compartment the funding between the subsidised and contributory group to protect the benefit for the poor people. However, it is unlikely to solve the inequity issue, as it is likely to further weakening the viability of JKN programme as a single payer. When the risk pooling is unable to sustain the increased demand from the contributory group, the restriction of JKN medical benefit and rising premium is inevitable. The healthier enrollees will discontinue their membership leaving the JKN programme with sicker enrollees who will keep contribute to rising costs. This cycle will continue which may lead to the collapse of JKN programme leaving the non-poor people working in informal sectors uninsured. Rather, we suggest that policymakers should explore other policy tools to expand the risk pooling and consider strategic purchasing to contain the healthcare costs.

## Electronic supplementary material

Below is the link to the electronic supplementary material.

Supplementary material 1 (DOCX 38 kb)
