# Supplementary material for: The impact of public health insurance on healthcare utilisation in Indonesia: evidence from panel data
Source: Int J Public Health. 2019 Feb 8;64(4):603–13. doi: 10.1007/s00038-019-01215-2 (PMC6517357; doi:10.1007/s00038-019-01215-2)
Supplement: Supplementary file 1 — Supplementary material 1 (DOCX 38 kb) [file 38_2019_1215_MOESM1_ESM.docx]

**The impact of public health insurance on healthcare utilisation in Indonesia: Evidence from panel data**

International Journal of Public Health

# Electronic Supplementary materials

**Online Resource 1. Summary of main sample from Indonesian Family Life Survey (IFLS), Indonesia 2007 and 2014**

**2007**

29,014 individuals

**2014**

22,711 individuals

6,303 individuals

982 contributory group*

2503 subsidised group*

8,576 Uninsured

10,650 Excluded**

1,440 reported dead in 2014

994 did not complete individual questionnaires

3,824 were lost to follow-up in 2014

45 partially completed individual questionnaires

*Both contributory and subsidised groups are enrolled in the Jaminan Kesehatan Nasional (JKN) programme

** Excluded group consists of individuals who were either a) previously uninsured in 2007 but became insured by non-JKN programme in 2014 or b) previously insured by any type of insurance in 2007 regardless their insurance status in 2014

**Online Resource 2. Sensitivity analysis of health insurance impact to different matching bandwidth for Kernel Epanechnikov, Indonesia 2007 and 2014**

|  | Probability of any outpatient care | Number of outpatient visits (total) | Number of outpatient visits (public) | Number of outpatient visits private | Probability of any inpatient care | Number of inpatient visits (total) | Number of inpatient visits (public) | Number of inpatient visits private |
| --- | --- | --- | --- | --- | --- | --- | --- | --- |
| **JKN Subsidised** | |  |  |  |  |  |  |  |
| 0.05 | 0.030 | 0.097** | 0.070** | 0.032 | 0.028*** | 0.038** | 0.030** | 0.013 |
|  | (0.021) | (0.045) | (0.032) | (0.025) | (0.010) | (0.016) | (0.013) | (0.010) |
| 0.005 | 0.030 | 0.104** | 0.072** | 0.034 | 0.028** | 0.039** | 0.030** | 0.014 |
|  | (0.024) | (0.048) | (0.035) | (0.028) | (0.012) | (0.018) | (0.014) | (0.011) |
| 0.001 | 0.031 | 0.111** | 0.069* | 0.040 | 0.029*** | 0.039* | 0.032* | 0.011 |
|  | (0.025) | (0.056) | (0.040) | (0.033) | (0.011) | (0.021) | (0.017) | (0.010) |
| **JKN Contributory** | |  |  |  |  |  |  |  |
| 0.05 | 0.075*** | 0.127 | 0.115*** | 0.040 | 0.084*** | 0.108*** | 0.073*** | 0.036*** |
|  | (0.018) | (0.067) | (0.026) | (0.053) | (0.012) | (0.018) | (0.012) | (0.012) |
| 0.005 | 0.077*** | 0.128** | 0.115*** | 0.043 | 0.085*** | 0.109*** | 0.072*** | 0.037*** |
|  | (0.019) | (0.058) | (0.022) | (0.051) | (0.013) | (0.019) | (0.013) | (0.011) |
| 0.001 | 0.080*** | 0.140 | 0.119*** | 0.050 | 0.087*** | 0.112*** | 0.072*** | 0.040*** |
|  | (0.020) | (0.074) | (0.028) | (0.064) | (0.013) | (0.019) | (0.014) | (0.011) |

*JKN = Jaminan Kesehatan Nasional/National Health Insurance.*

*The reported standard errors in parentheses were calculated by bootstrapping with 200 replications. Quintiles were determined based on assets index in 2007. Significance: * p<0.1; ** p<0.05; *** p<0.01*

**Online Resource 3. Medical conditions included in the construction of health status variables from Indonesian Family Life Survey (IFLS), Indonesia 2007 and 2014**

Number of acute conditions include:

1. Headache
2. Bloody cough
3. Difficulty breathing
4. Fever
5. Stomach ache
6. Nausea/vomiting
7. Diarrhoea
8. Swollen legs
9. Skin infection
10. Eye infection
11. Toothache
12. Cold sores

Number of chronic conditions include:

1. Hypertension
2. Diabetes
3. Tuberculosis
4. Asthma
5. Other lung conditions
6. Heart attack, coronary heart disease, angina, or other heart problems
7. Liver
8. Stroke
9. Cancer or malignant tumour
10. Arthritis/rheumatism
11. High cholesterol (Total or LDL)
12. Prostate illness
13. Kidney disease except for tumour or cancer
14. Stomach or other digestive diseases
15. Emotional, nervous, or psychiatric problems
16. Memory-related disease
